# *De Novo* Characterization of Fall Dormant and Nondormant Alfalfa (*Medicago sativa L*.) Leaf Transcriptome and Identification of Candidate Genes Related to Fall Dormancy

**DOI:** 10.1371/journal.pone.0122170

**Published:** 2015-03-23

**Authors:** Senhao Zhang, Yinghua Shi, Ningning Cheng, Hongqi Du, Wenna Fan, Chengzhang Wang

**Affiliations:** College of Animal Science and Veterinary Medicine, Henan Agricultural University, Zhengzhou, Henan 450002, China; Sabanci University, TURKEY

## Abstract

Alfalfa (*Medicago sativa* L.) is one of the most widely cultivated perennial forage legumes worldwide. Fall dormancy is an adaptive character related to the biomass production and winter survival in alfalfa. The physiological, biochemical and molecular mechanisms causing fall dormancy and the related genes have not been well studied. In this study, we sequenced two standard varieties of alfalfa (dormant and non-dormant) at two time points and generated approximately 160 million high quality paired-end sequence reads using sequencing by synthesis (SBS) technology. The *de novo* transcriptome assembly generated a set of 192,875 transcripts with an average length of 856 bp representing about 165.1 Mb of the alfalfa leaf transcriptome. After assembly, 111,062 (57.6%) transcripts were annotated against the NCBI non-redundant database. A total of 30,165 (15.6%) transcripts were mapped to 323 Kyoto Encyclopedia of Genes and Genomes pathways. We also identified 41,973 simple sequence repeats, which can be used to generate markers for alfalfa, and 1,541 transcription factors were identified across 1,350 transcripts. Gene expression between dormant and non-dormant alfalfa at different time points were performed, and we identified several differentially expressed genes potentially related to fall dormancy. The Gene Ontology and pathways information were also identified. We sequenced and assembled the leaf transcriptome of alfalfa related to fall dormancy, and also identified some genes of interest involved in the fall dormancy mechanism. Thus, our research focused on studying fall dormancy in alfalfa through transcriptome sequencing. The sequencing and gene expression data generated in this study may be used further to elucidate the complete mechanisms governing fall dormancy in alfalfa.

## Background

Alfalfa (*Medicago sativa* L.) is one of the most widely cultivated perennial forage legumes in the world and is prized for its great yield potential, high nutritional value, wide adaptation and as a biofuel feedstock [1–3]. Alfalfa is a very important crop for sustainable agriculture because of its ability to fix nitrogen through association with rhizobia[4]. The leading countries for alfalfa production are the United States (US), Argentina, Canada, Russia, Italy and China[5]. Alfalfa hay products with a value more than $10 billion were harvested from 7 million hectares in 2012 in the US (USDA National Agricultural Statistics Service, 2012). Some alfalfa varieties cannot survive through the harsh winter in specific regions, whereas others survive but with reduced biomass accumulation than those cultivated in temperate regions[6]. This phenomenon is related to an important character in alfalfa, fall dormancy (FD), which is induced by the shortening photoperiod and falling temperatures in late summer or early autumn[6]. Dormant alfalfa varieties are ensured a better survival in harsh winter, but with lesser biomass accumulation. Plant height is the only index indicating the FD in alfalfa. There are eleven fall dormancy classes (FDC) at present classified on the basis of the regrowth height after cutting at a special autumn period[7]. These eleven classes can be broadly divided into dormant (FDC 1–4), semidormant (FDC 5–7) and non-dormant categories (FDC 8–11)[8]. Dormant alfalfa varieties reduce or cease shoot growth and produce short and decumbent shoots after cutting in the autumn. Non-dormant varieties continue rapid shoot regrowth and produce tall upright shoots in the autumn. Semidormant varieties show phenotypes intermediate between dormant and non-dormant alfalfa[3,8].

The physiological, biochemical and molecular mechanisms causing FD are not fully understood. Our previous study showed that the photoperiod was more important than temperature in causing FD in alfalfa[9]. Other studies showed that there was a positive correlation between fall growth and winter injury in alfalfa[10–13]. Moreover, sugar content of roots and sugar content of crown buds in alfalfa were closely related with winter hardiness[14–18]. Winter injury was found to be negatively associated with carbohydrate content especially the hexosan content in alfalfa roots[19]. Increased levels of sucrose, stachyose and raffinose, and reduced levels of glucose, fructose and starch levels were related to freezing tolerance[16]. These studies were mainly concerned with the effects of temperature on FD in alfalfa. Thus, new methods are required to understand the mechanism of FD. The advent of next-generation sequencing (NGS) technology [20,21] has facilitated the use of genomic resources of non-model species at a low cost. Several studies including single nucleotide polymorphism (SNP) discovery[22–24], gene identification and transcript profiling [23] have recently been done in alfalfa. However, the genome sequence of this autotetraploid, allogamous and heterozygous species [25] is not available at present. The genome of alfalfa was predicted to be 800 Mb in size[24].

In this study, we used NGS technology to sequence the leaf transcriptome of alfalfa (including dormant and non-dormant types, in May and September) using leaf tissues. The objectives of this study were to: (i) sequence and annotate the leaf transcriptome of alfalfa, (ii) identify differentially expressed genes between dormant and non-dormant varieties at two different time points in alfalfa. These results would provide a valuable genomic resource for further studies in FD in alfalfa.

## Materials and Methods

### Ethics Statement

The field study was conducted at the Experimental Station of Henan Agricultural University, Zhengzhou, China. All plant materials collected in the field study were not protected species.

### Plant materials and growth condition

Alfalfa standard varieties Maverick (FDC1) and CUF101 (FDC9) were introduced from the United States and planted on sandy loam soil at the Experimental Station of Henan Agricultural University, Zhengzhou, China (34°19×N, 113°35×E). The region had on average, 614 mm of annual precipitation, 14.6°C of mean air temperature with extremes of -10.2°C in January and 41.5°C in July over the past 25 years. The soil was a Hapludolls sandy loam and contained (0–30 cm depth) 14 g/kg of OM, 0.94 g/kg of total N, 63.5 mg/kg of alkaline-N, 10.8 mg/kg of Bray-P, 127 mg/kg of test K with pH 7.5 (1:1 in water). The field was in an alfalfa-alfalfa-alfalfa-corn (*Zea mays*)-winter wheat (*Triticum aestivum* L.) cropping system before the current study. The field was considered sufficient in K, but had shortage of N and P before the current study, therefore, 81 kg/ha of N as urea and 96 kg/ha of P_2_O_5_ as calcium phosphate were applied and incorporated into 0 to15 cm depth during the land preparation for the study.

Each plot was 10.0 m long and consisted of five rows of alfalfa with 0.6 m row spacing, seeded by hand on October 1, 2009. The seedlings were thinned to 4 plants/m of linear row. The seeds were not scarified or inoculated, because, in the previous two years; the land had been cropped to alfalfa. Due to the occurrence of severe drought from the spring in 2010, irrigation was provided in early March of that year. Fertilizer was not applied during growth periods. Weed control was performed by hand or hoeing, and insects were controlled as required.

Thirty randomly selected plants were measured for plant natural height at 14 days after cutting in each plot and the mean of height was calculated for each plot. Alfalfa leaves were collected from Maverick and CUF101, both on May 19 and September 23, 2011, fourteen days after alfalfa was cut. Leaves were immediately frozen in liquid nitrogen, and then stored at -80°C. We used abbreviations D5, ND5, D9 and ND9 for dormant type (Maverick) in May, non-dormant type (CUF101) in May, dormant type in September and non-dormant type in September, respectively.

### RNA extraction, cDNA library preparation and sequencing

Total RNA was isolated from eight samples [alfalfa leaves, (D5, ND5, D9, ND9) × (two biological replicates)] using TRIzol Reagent (Invitrogen, Carlsbad, CA, USA) according to the manufacturer’s instruction. Genomic DNA contamination was removed from total RNA using RNase-free DNase I (Fermentas, Vilnius, Lithuania). RNA samples were quantified using Qubit 2.0 Fluorometer (Life Technologies, Carlsbad, CA, USA) and the RNA integrity (RIN) was checked with the RNA6000 Nano Assay using the Agilent 2100 Bioanalyzer (Agilent Technologies, Palo Alto, CA, USA). cDNA library preparations and sequencing reactions were conducted at GENEWIZ, Inc. (South Plainfield, NJ, USA). The samples were sequenced using a 2×100 paired-end (PE) configuration; 101 cycles were sequenced from each end of the libraries. Image analysis and base calling were conducted using the HiSeq Control Software (HCS) on HiSeq 2000. These base calls were used to generate BCL files using Illumina’s CASAVA 1.8.2 program. The resulting BCL files were subsequently converted to FASTQ files and sequence reads that passed Illumina’s standard filtering were retained for further analysis using Illumina’s CASAVA 1.8.2 program.

### *De novo* transcriptome assembly

The sequencing adapters/primers were trimmed. The quality of these sequencing reads was assessed using the FastQC tool[26]. *De novo* transcriptome was performed on a virtual private server with 24 cores and 196 GB of memory. The PE reads of eight samples (160 million sequence reads) were assembled as one dataset using Trinity (Version r2012-05-18) [27]. Trinity uses the *de Bruijn* graph strategy to assemble the transcriptome; it was run on the PE reads with a fixed *k*-mer value of 25, *k*-mer method of Jellyfish, max memory of 220 GB, 32 CPUs, a max butterfly HeapSpace of 12 GB, the amount of RAM initially each thread would be in the butterfly job of 2 GB and CPUs used for butterfly of 16. To assess the completeness of the *de novo* assembled transcriptome, Core Eukaryotic Genes Mapping Approach (CEGMA; Version 2.4.010312) program [28] was used. Open reading frames (ORFs) were identified using TransDecoder (http://transdecoder.sourceforge.net; Version rel16JAN2014) program, with default parameters. All Illumina raw sequence reads have been deposited in the NCBI Sequence Read Archive (SRA) under accession number SRA057663. The *de novo* assembled transcriptome data has been deposited at DDBJ/EMBL/GenBank under the accession GAFF00000000. The version described in this paper is the first version, GAFF01000000.

### Similarity analysis and functional annotation

Fifteen plant proteome data sets from completely sequenced genomes were downloaded from three different file transfer protocol (FTP) sites for sequence conservation analysis. Among these proteome data sets, sequences for *Arabidopsis thaliana*, *Brachypodium distachyon*, *Carica papaya*, *Cucumis sativus*, *Glycine max*, *Malus domestica*, *Medicago truncatula*, *Oryza sativa*, *Physcomitrella patens*, *Populus trichocarpa*, *Sorghum bicolor*, *Vitis vinifera* and *Zea mays* were downloaded from Phytozome (ftp://ftp.jgi-psf.org/pub/compgen/phytozome/v9.0/; Version 9.0). *Lotus japonicus* proteome was downloaded from Institute of Bioinformatics and Systems Biology, Helmholtz Zentrum Munchen German Research Center for Environmental Health (ftp://ftpmips.helmholtz-muenchen.de/plants/lotus/). *Jatropha curcas* proteome was downloaded from Kazusa DNA Research Institute (ftp://ftp.kazusa.or.jp/pub/jatropha/). BLASTX search against these fifteen proteome data sets was done using NCBI BLAST+ 2.2.26 with an E-value cut-off of 1E-05.

We used BLASTX search against NCBI non-redundant (NR) protein database for leaf transcriptome annotation with an E-value cut off of 1E-05. For speeding the annotation process, only the first five hits per sequence were retained. The BLASTX xml result was imported into Blast2GO to get gene ontology (GO) annotation. The leaf transcriptome was also submitted to Kyoto Encyclopedia of Genes and Genomes (KEGG) Automatic Annotation Server (KAAS; http://www.genome.jp/kaas-bin/kaas_main; Version 1.67x) to retrieve the KEGG orthology (KO) assignments and KEGG pathways, using single-directional best hit (SBH) assignment method.

Transcription factor (TF) families were identified using the hidden Markov model (HMM) profiles generated from domain alignments from 84 gene families in Plant Transcription Factor database (PlnTFDB; http://plntfdb.bio.uni-potsdam.de/v3.0/)[29]. Three programs in HMMER suit (Version 3.0b3) were used: *hmmbuild*, *hmmpress* and *hmmscan*[30].

### GC content analysis and simple sequence repeat (SSR) markers identification

GC content analysis was performed using publically available Perl scripts. MIcroSAtellite identification tool (MISA; http://pgrc.ipk-gatersleben.de/misa/) was used to identify SSR[31]. Mononucleotide repeats of at least ten times, dinucleotide repeats of at least six times, and trinucleotide, tetranucleotide, pentanucleotide and hexanucleotide repeats of at least five times were searched for SSRs. Maximal number of bases interrupting two SSRs in a compound microsatellite was 100. SSR primers were developed using MISA and Primer3 (http://primer3.sourceforge.net/; Version 2.3.6).

### Mapping of sequence reads back to transcriptome and expression analysis

The sequence reads from eight samples were aligned to the assembled transcriptome individually, using the Perl script that comes with the Trinity assembler with the aligner bowtie[32]. The resulting files were then quantified using Trinity-supplied RSEM [33] with default parameters to get gene based raw hit-count data. The gene based raw results were formatted into comma separated values (CSV) text files and uploaded into Simbiot platform[34]. Then the raw-hit count data was transformed using DESeq [35] variance reduction algorithm. The transformed data matrixes were then normalized using a quantile [36] algorithm built into the Bioconductor [37] package Limma [38] with a default option. Expression analysis was performed using Limma with Benjamini-Hochberg false discovery control procedure[39]. The gene expression results with at least 2-fold change and adjusted *P* value ≤ 0.01 were considered as significantly differentially expressed genes using R software[40]. GO enrichment analyses were studied using BiNGO plugin in Cytoscape (Version 3.0.2), with the Benjamini & Hochberg False Discovery Rate (FDR) correction and the significance level of 0.05.

### Validation of differentially expressed genes

Total RNA was isolated using TRIzol Reagent (Invitrogen) according to the manufacturer’s recommendation. After RNase-free DNase I (Fermentas) treatment, the first strand cDNA for each sample was made from 1 μg total RNA using RevertAid First Strand cDNA Synthesis Kit (Fermentas) following the manufacturer’s instruction and diluted 10x before performing PCR. A total of 22 differentially expressed genes were randomly selected to get the quantitative PCR (qPCR) validation. All qPCR primers were picked using the web-based Primer3Plus with the built-in special qPCR settings[41]. *Medicago sativa* putative glyceraldehyde-3-phosphate dehydrogenase (Ms*GAPDH*) was selected as the reference gene[42]. Primer sequences used in this study are shown in S1 Table. Three independent biological replicates for each condition, and three technical replicates for each biological replicate were analyzed using qPCR analysis on a Mastercycler ep realplex 2 (Eppendorf AG, Hamburg, Germany) following the manufacturer’s recommendations. qPCR was performed in a total of 15 μL reaction volume containing 7.5 μL Maxima SYBR Green qPCR Master Mix (2X); ROX Solution provided (Fermentas), 0.2 μL forward primer (10 ng/μL), 0.2 μL reverse primer (10 ng/μL), 5.1 μL nuclease-free water and 2 μL of template cDNA. The PCR conditions were: 10 minutes of the initial denaturation at 95°C, 40 cycles of 15 seconds at 95°C and 60 seconds at 60°C, followed by steps for dissociation curve generation (15 seconds at 95°C, 15 seconds at 60°C and 15 seconds at 95°C). Data were collected, and quantification cycle (Cq) or threshold cycle (Ct) values were analyzed using 2^-ΔΔCt^ method.

## Results and Discussion

### Natural height of plant materials used in our study

Natural plant height is the only index for FD evaluation at present[7]; therefore, to determine if these alfalfa varieties showed FD morphology or not, plant height of 30 randomly selected plants from both Maverick and CUF101 was measured in both May and September (see Materials and Methods section) (Table 1). According to the plant height shown in Table 1, there was no significant difference in height between D5 and ND5 in May, suggesting that long days are not for FD. There was no significant difference in plant height of the dormant and non-dormant varieties between May and September (ND5 and ND9) suggesting that FD induction was not sensitive to long day/short day period and different temperatures. For dormant types, there was a significant difference in regrowth height between D5 and D9 indicating that, FD was induced by seasonal change. In September, there was a significant difference between D9 and ND9 indicating that D9 showed FD morphology while ND9 did not, which could be attributed to the characteristics of dormant and non-dormant alfalfa themselves. Teuber (1998) showed that FD was measured using a scale of 5 cm increment in regrowth height [7] indicating that D9 had entered the stage of FD while others had not.

**Table 1:** Natural height of plants selected for sequencing.

**Variety**	**Height on May 19 (cm)**	**Height on September 23 (cm)**
Maverick	28.47±1.11	7.83±0.87
CUF101	29.20±1.40	27.53±0.90

Plant height was measured using Mean±SD (standard deviation).

### Transcriptome sequencing and *de novo* assembly

Transcriptome profiles were made from mRNA extracted from alfalfa leaves of D5, ND5, D9 and ND9. Two biological replicates were used for each sample to generate a total of eight cDNA libraries for RNA-seq using an Illumina HiSeq 2000. Approximately 20 million high-quality paired-end (PE) (2×100 bp) reads were generated for each sample by sequencing, i.e.160 million in total, which is approximately 32.32 billion bases. An average of 38 Phred quality score per read was achieved, indicating a “high quality” for all sequencing reads. Due to the lack of a reference genome or transcriptome for alfalfa, all sequencing reads were put together to do a *de novo* transcriptome assembly using Trinity[27], and this leaf transcriptome was used as the standard. Trinity *de novo* assembly utilizes algorithms based on *de Bruijn* graphs. It focuses on analysis of short words (*k*-mers), with every *k*-mer represented as a unique node, regardless of how many times it appears in the data. This significantly simplifies the complexity of handling very large data sets from NGS and accommodates reads of variable length. In the assembly process, results from low quality reads that do not connect to any other read on the graph, from reads that contain errors in the middle of the sequence and from reads that accidentally overlap were removed by examining the graph and its links. Additional corrections and heuristics such as noise reduction, prediction of full-length fragments and handling of alternative splicing were performed to ensure the quality of the assembled leaf transcriptome.

**Fig 1:**
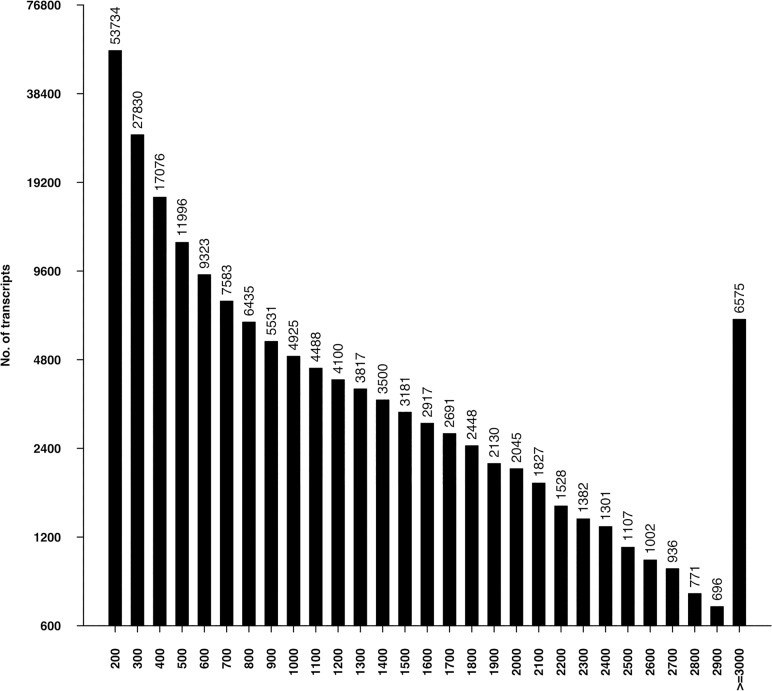
Sequence length distribution of alfalfa leaf transcriptome. Lenth of the 192,875 transcripts in the alfalfa leaf transcriptome were illustrated. The length ranges from 200 bp to more than or equal to 3,000 bp.

From the *de novo* transcriptome assembly, we detected a total of 192,875 putative transcripts assembled from a total of 160 million sequence reads, representing 165,103,228 bp (165.1 Mb) of the sequence. This assembly had an average transcript length of 856 bp and N50 value of 1,451 bp with the transcript length ranged from 201 bp to 15,740 bp. Approximately 53,367 (27.67%) of the transcripts were at least 1,000 bp (Table 2). Only a small fraction (6,575) of the transcripts were more than or equal to 3,000 bp. Length distribution of alfalfa leaf transcriptome was depicted in (Fig. 1). The average length of the transcripts was higher than those of other alfalfa transcriptome sequencing projects of Yang *et al*. (284 bp)[23], Han *et al*. (541 bp) [22]and Postnikova *et al*. (348 bp)[43], but lower than that of Li *et al*. (1,065 bp)[24]. The isoforms were represented by 94,700 components, a loose representation of putative genes. The isoforms’ distribution is shown in S1 Fig. The completeness of the *de novo* assembled transcriptome was assessed using CEGMA pipeline to compare our 192,875 transcripts to the 248 ultra-conserved eukaryotic genes (CEGs) defined by CEGMA. It showed that 237 of the 248 (95.56%) CEGs were present “in full” and 244 (98.39%) were present “in partial”, indicating that the *de novo* assembled transcriptome was sufficiently complete[44–47]. In all of the 192,875 transcripts, 112,842 candidate ORFs were found using TransDecoder with default parameters. These ORFs corresponded to 88,327 peptide sequences for the final candidate ORFs (all shorter candidates within longer ORFs were removed).

**Table 2:** Summary of *de novo* assembled alfalfa leaf transcriptome.

**Data type**	**Number**
Total sequence	192,875
No. of sequences ≥ 1000 bp	53,367
Total bases	165,103,228
No. of bases ≥ 1000 bp	105,827,064
Maximum sequence (bp)	15,740
Minimum sequence (bp)	201
Avg. length (bp)	856
Median length (bp)	484
N50 length (bp)	1,451
GC (%)	37.84

Our results indicate that short PE reads can be effectively assembled and used to characterize the gene expression analysis of non-model organisms. Although the genome of alfalfa is unavailable at present, the genome size was predicted to be 800 Mb[24]. Alfalfa leaf transcriptome sequencing in our study resulted in a relatively high coverage depth (40x). The resulting 192,875 putative transcripts generated in *de novo* transcriptome assembly were used as a basis for gene expression analysis. Mapping the sequence reads back to the assembled transcriptome resulted in about 70% of reads that were mapped back on the assembled transcripts (Table 3). The datasets were further analyzed using RSEM [33] and resulted in about 20% of the putative isoforms with more than 10 reads mapped across all the samples (Table 3).

**Table 3:** Statistics of sequence reads mapping back to alfalfa leaf transcripts.

**Samples**	**Reads aligned**	**Number of transcripts with reads mapped**		
		**0 read**	**1–10 reads**	**> 10 reads**
D5-rep1	13,802,849 (69.0%)	99,864	52,815	39,395
D5-rep2	14,209,306 (71.0%)	102,982	49,614	39,602
ND5-rep1	14,021,469 (70.1%)	105,299	48,645	38,266
ND5-rep1	14,285,151 (71.4%)	99,817	51,019	41,287
D9-rep1	13,798,547 (69.0%)	105,210	48,281	38,674
D9-rep2	13,545,778 (67.7%)	103,250	48,996	39,906
ND9-rep1	13,872,852 (69.4%)	101,160	50,666	40,361
ND9-rep2	13,753,460 (68.8%)	96,842	52,756	42,531

Each sample had 20 million paired-end reads. About 70% of reads that mapped back on the assembled transcripts. rep1 and rep2 represent the two biological replicates in each sample.

### Sequence similarity and functional annotation of alfalfa leaf transcriptome

**Fig 2:**
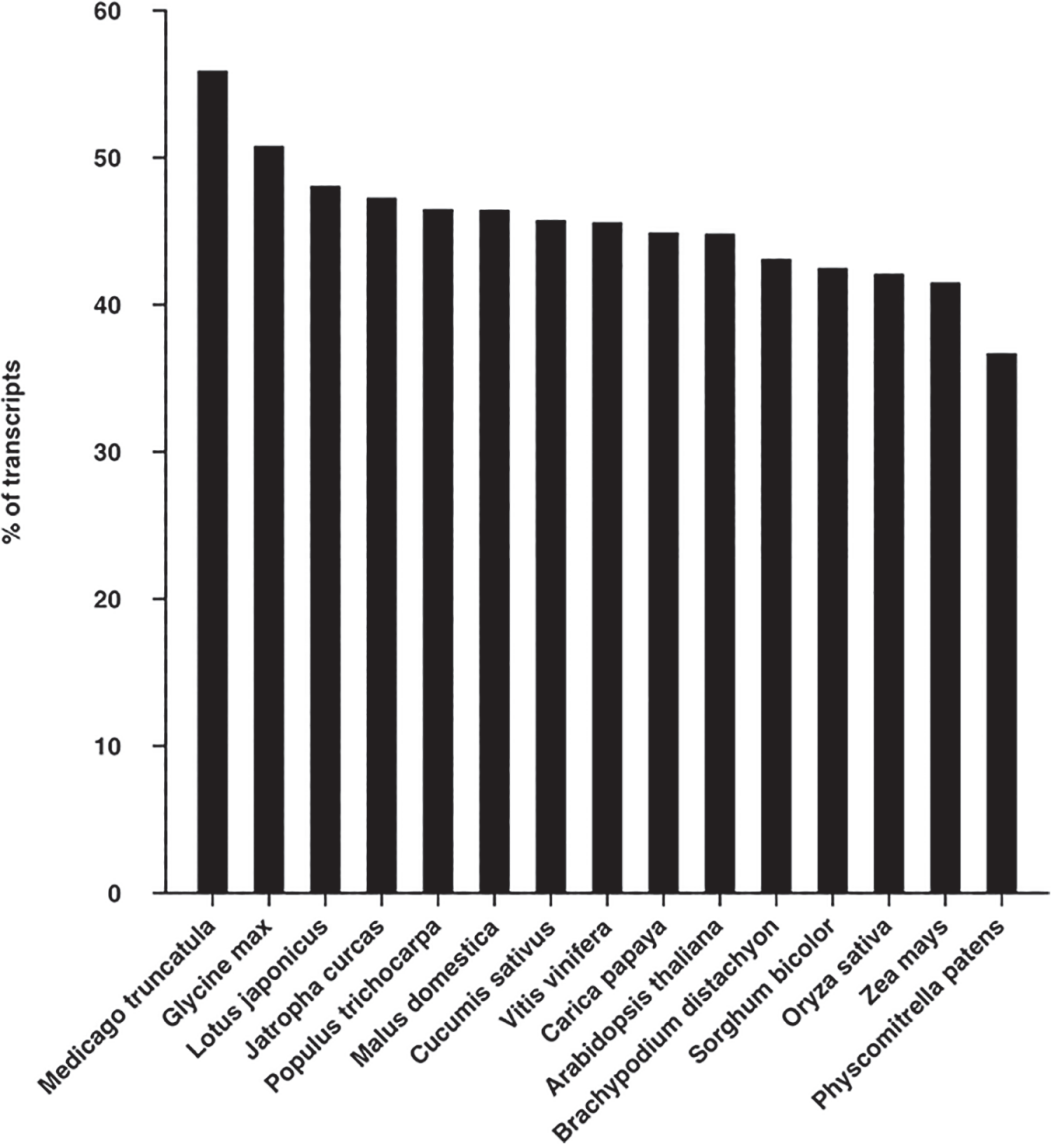
Similarity of alfalfa leaf transcriptome with proteomes of fifteen sequenced plants. It was performed using BLASTX with an E-value threshold of ≤ 1E-05.

**Fig 3:**
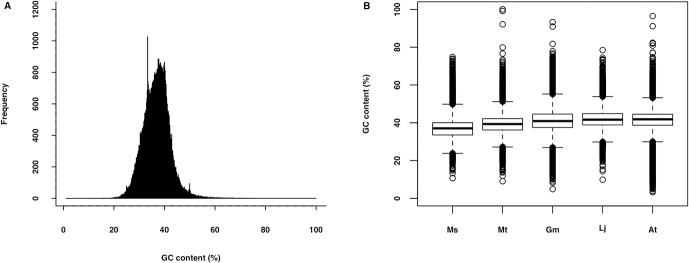
GC content analysis of alfalfa leaf transcripts. A, Frequency of GC content of alfalfa leaf transcripts. B, Distribution of GC content of transcripts for alfalfa (*Medicago sativa*; Ms), *Medicago truncatula* (Mt), *Glycine max* (Gm), *Lotus japonicus* (Lj) and *Arabidopsis thaliana* (At). The box shows the interquartile range. The circles are outliers of the GC content.

Sequence similarity between the alfalfa leaf transcriptome and fifteen species of other plants whose genomes have been completely sequenced was analyzed by utilizing BLASTX search against proteomes of these plants, with an E-value of 1E-05 as a cut-off. The BLASTX search results showed that about 107,730 (55.85%) of alfalfa leaf transcripts showed significant similarity to *Medicago truncatula* proteins as expected, followed by *Glycine max* (50.74%), *Lotus japonicus* (48.02%), *Jatropha curcas* (47.21%) and the smallest proportion (70,672; 36.64%) showed significant similarity to *Physcomitrella patens* (Fig. 2). Overall, 115,343 (59.80%) of alfalfa leaf transcripts exhibited significant similarity with at least one protein sequence from the fifteen plant proteomes. These conserved transcripts from other plant species might perform similar functions in alfalfa. The overall similarity was lower than that in the chickpea transcriptome[48]. The low similarity with other plants may be due to the deep coverage of the transcriptome resulting in a higher percentage of non-coding sequences, or heteronuclear transcripts that contain too many introns to accurately produce BLAST hits. We used alfalfa leaves as research objects to sequence the alfalfa leaf transcriptome, part of the transcripts might be leaf-specific and thus lowered the overall similarity. The transcriptome generated in this study had approximately 60.83% of transcripts with GC content ranged from 35%-45% (Fig. 3A). The GC content of the alfalfa transcriptome (37.84%) was similar to that of *Medicago truncatula* (39.45%), but lower than that of *Arabidopsis thaliana* (42.19%), *Glycine max* (41.52%) and *Lotus japonicus* (42.37%). The GC content may be related to the variation of mutation bias; thermo stability [49] and gene coding regions[50]. The range of GC content of these species were also depicted (Fig. 3B).

**Fig 4:**
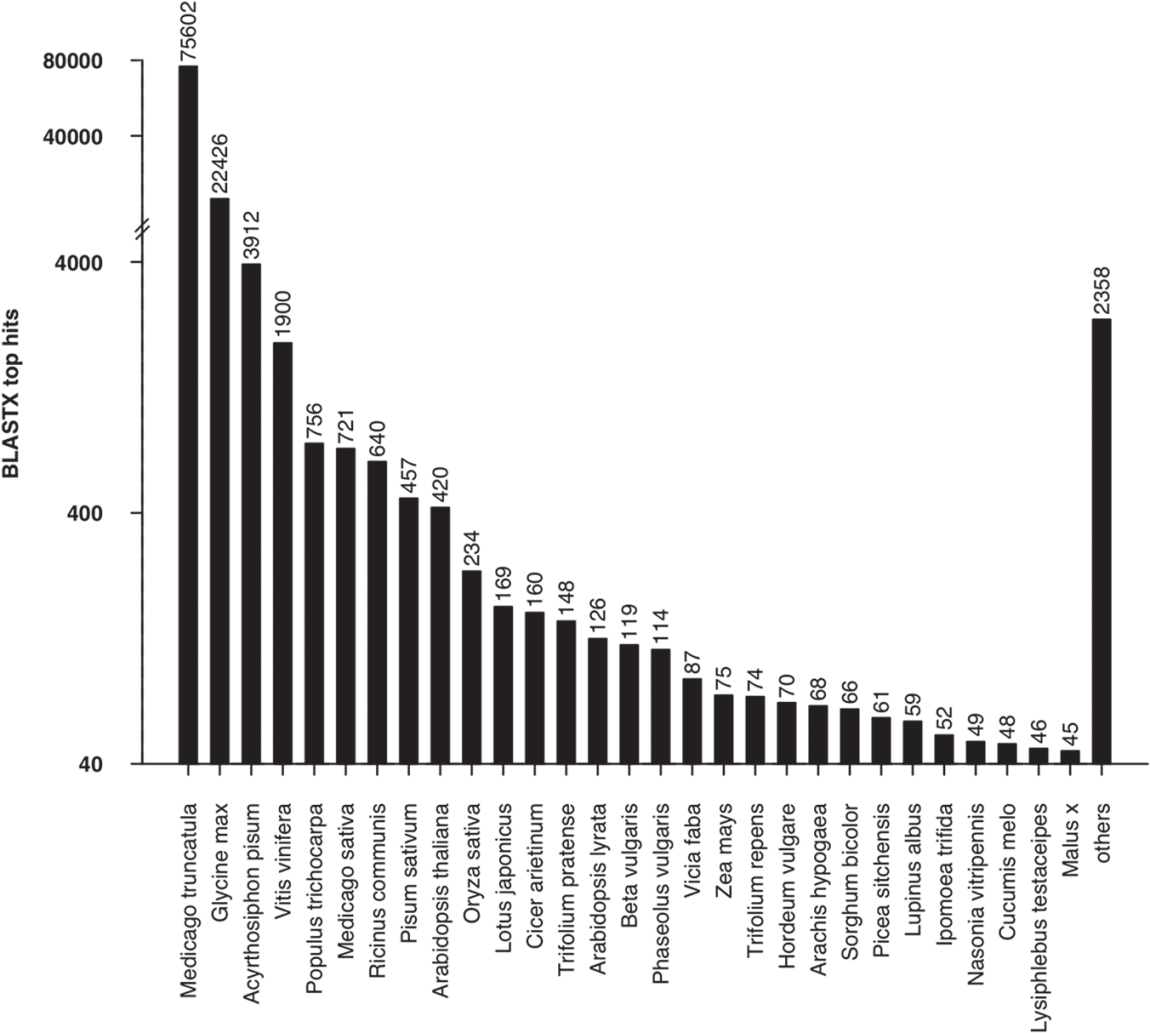
Species distribution of BLASTX top hits against NCBI NR database. Alfalfa leaf transcriptome was subjected to NCBI NR database for sequence similarity search using BLASTX (E-value threshold of ≤ 1E-05). Values above bars show the number of transcripts hit in each species.

**Fig 5:**
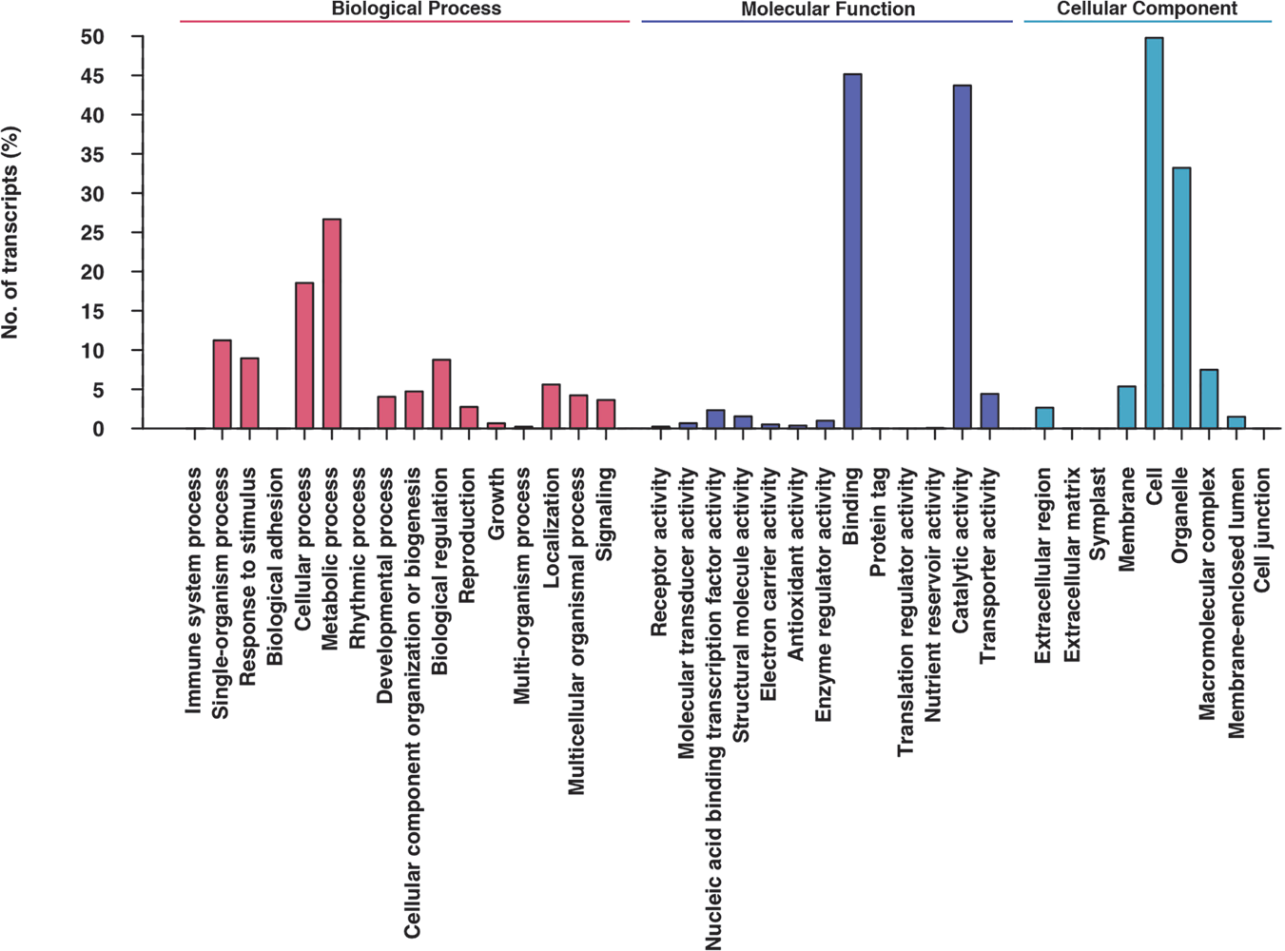
Gene Ontology (GO) distribution of alfalfa leaf transcripts at level 2. GO Slim terms were assigned to 77,307 transcripts and categorized into three groups: biological process (58,412), molecular function (63,602) and cellular component (42,472).

**Fig 6:**
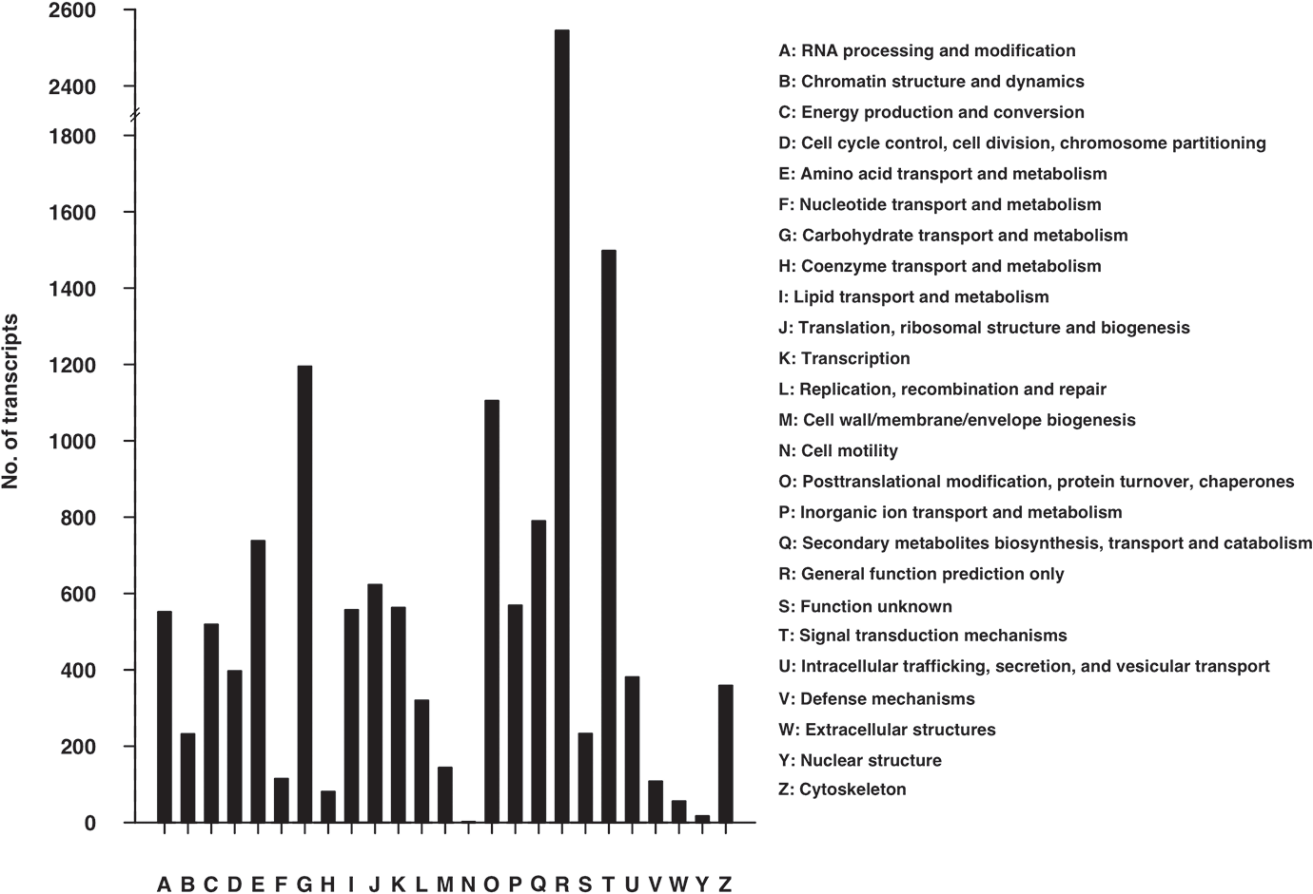
EuKaryotic Orthologous Groups (KOG) classifications of alfalfa transcripts. Of transcripts annotated, 13,699 were classified into 25 KOG categories.

Alfalfa leaf transcriptome annotation was carried out against NCBI NR database using BLASTX with an E-value cut-off of 1E-05. A total of 111,062 transcripts showed significant hits with the NR database. The BLASTX top-hit species distribution of the 111,062 transcripts showed the highest hits to *Medicago truncatula* (75,602; 68.07%), followed by *Glycine max* (22,426; 20.19%) and *Acyrthosiphon pisum* (3,912; 3.52%) (Fig. 4). The low annotation rates might due to the BLASTX process, the frame will not be known a total of three different translations are prepared and those that do not contain an open reading frame are discarded for many transcripts; only the best match was recorded as a possible annotation. Otherwise, autotetraploid and allogamous of alfalfa might be the other key factor. Further more, Blast2GO [51] suite was used to retrieve GO [52] terms using the annotation result of BLASTX against NR. Among these, 111,062 annotated transcripts, 77,307 transcripts had at least one GO term. Based on GO-Slim terms, these transcripts were categorized into three groups, Biological Process (BP), Molecular Function (MF) and Cellular Component (CC) (Fig. 5). As a result, 63,602 transcripts were grouped under MF, followed by 58,412 under BP and 42,472 under CC group. In the MF group, 45.15% of transcripts were associated with binding, followed by catalytic activity (43.71%) and transporter activity (4.41%). In the BP group, 26.66% of transcripts belonged to metabolic process, followed by cellular process (18.55%), single-organism process (11.23%) and response to stimuli (8.94%). Under the CC group, the largest proportion of transcripts was involved in the cell (47.78%), followed by the organelle (33.21%), macromolecular complex (7.48%) and membrane (5.35%) (Fig. 5). The GO results were similar to *Camellia sinensis* [53] and *Daucus carota* var. *sativus* L.[54]. The annotated sequences were functionally classified using EuKaryotic Orthologous Groups (KOG)[55], which resulted in 13,699 matching 25 KOG clusters (Fig. 6). KOG classification showed that the assembled transcriptome had 25 categories, indicating the landscape of alfalfa transcriptome. The largest part was ‘general function prediction only’ as can be noted from other studies[56–59], followed by ‘signal transduction mechanisms’ and ‘carbohydrate transport and metabolism’ that may be related to the experimental materials we collected, leaves. As FD was affected by photoperiod and temperature sugar accumulation was found during FD, the ‘signal transduction mechanisms’ and ‘carbohydrate transport and metabolism’ may play an important role in FD in alfalfa. KAAS [60] web server was used to identify different pathways of transcriptome generated in our experiment. Only 30,165 (15.64%) of all transcripts in the alfalfa leaf transcriptome were assigned to 3,474 KO ids, which were categorized into 323 KEGG [61] pathways. The ‘Metabolic pathways’ was the largest pathway with 6,718 transcripts associated with it, followed by ‘Biosynthesis of secondary metabolites’ and ‘Microbial metabolism in diverse environments’ pathways associated with 3,403 and 1,447 transcripts, respectively (S2 Table). The KAAS analysis enabled us to access a valuable resource to investigate pathways of alfalfa relate to FD. The number and assortment of allocated GO categories and KEGG pathways provide a good indication of the large diversity of expressed genes from the leaf transcriptome in alfalfa[62].

### Identification of TFs and SSR

**Fig 7:**
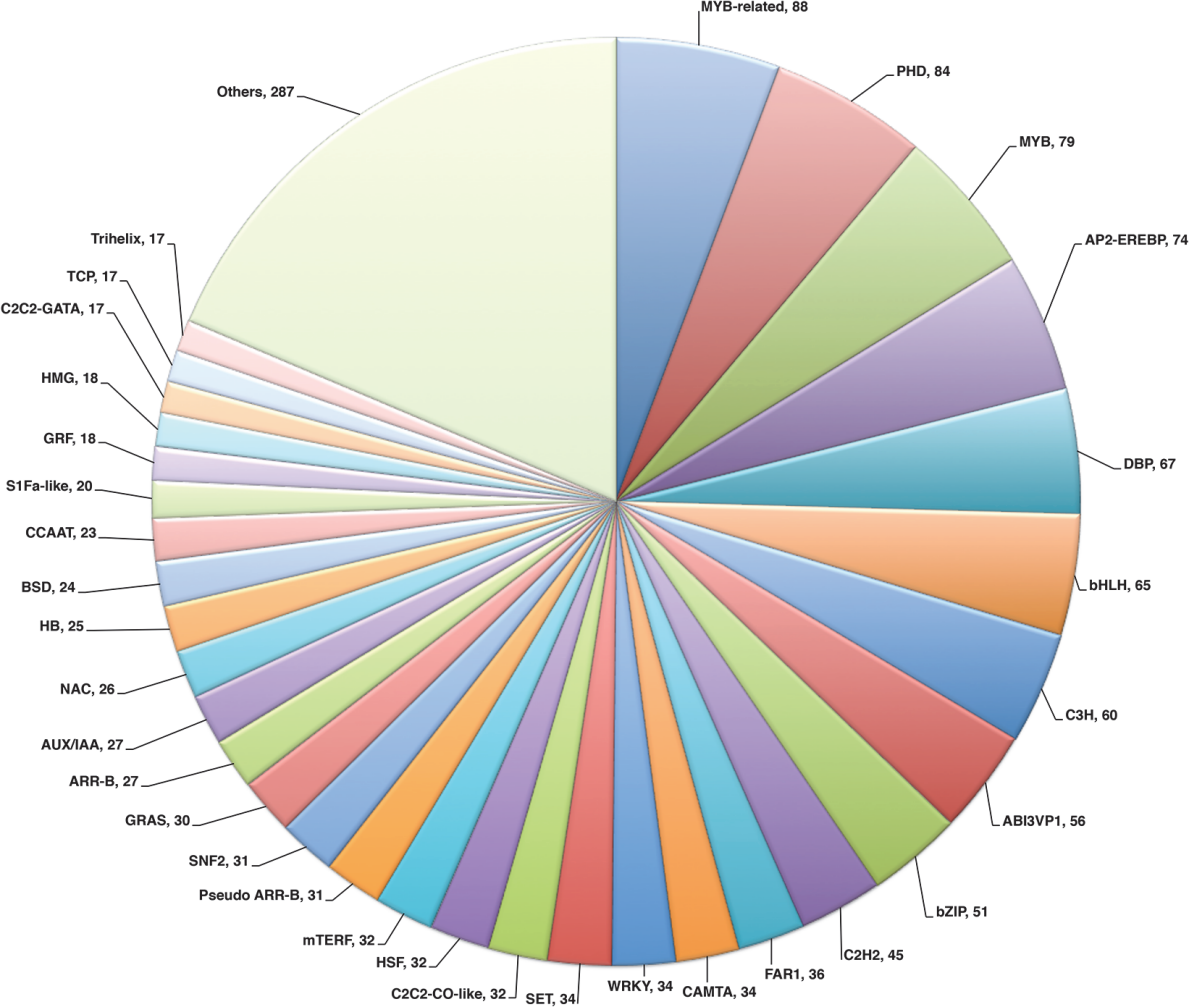
Transcription factor families identified in alfalfa leaf transcriptome. Transcription factors were identified using BLASTX with an E-value threshold of ≤ 1E-05 against the PlnTFDB. A total of 1,541 transcription factors were identified in 81 transcription factor families.

TFs are proteins that bind specifically to DNA sequences to control gene expression[63], which play an important role in regulating plant gene expression, development and responses to stimuli or stress[64]. In our study, a total of 1,541 (0.8%) TFs were identified in alfalfa leaf transcriptome, these TFs distributed in 1,350 transcripts, which belong to 81 of the total of 84 TF families available in PlnTFDB [29] we searched. The number of transcripts belonging to TF families varied from 1 to 88 (5.7%) in alfalfa transcripts. The largest TF family was the MYB-related (88; 5.71%), followed by PHD (84; 5.45%), MYB (79; 5.13%), AP2-EREBP (74; 4.80%), DBP (67; 4.35%), bHLH (65; 4.22%), C3H (60; 3.89%), ABI3VP1 (56; 3.63%) and bZIP (51; 3.31%) (Fig. 7). Comparisons of major TF families with *Arabidopsis* and *Medicago truncatula* are shown in S2 Fig. As S2 Fig. shows, the conservation of some TF families indicated the presence of conserved gene regulatory mechanisms in alfalfa leaves. Horvath [65]showed that a mutation caused by deletion of a locus containing several tandomly duplicated MADS-box TFs prevents dormancy induction in peach[66].

SSRs or microsatellite markers are tandem DNA repeats of 1 to 6 bases[67], which have been widely used in plant materials[68–70]. After the analysis using Perl script MISA[31], a total of 41,973 SSRs were identified in 33,691 (17.5%) transcripts of alfalfa. The average frequency of SSRs in alfalfa transcripts was one SSR per 3.93 kb of alfalfa leaf transcriptome sequence. Among these SSRs identified, 6,341 (3.3%) transcripts contained more than one SSR, and 2,716 (1.4%) SSRs were present in compound formation. The largest proportion of SSRs identified was the mononucleotide (66.7%), followed by trinucleotide (18.5%) and dinucleotide (13.5%). The trend was consistent with the results reported for chickpea[48]. A small number of tetranucleotide (510), pentanucleotide (35) and hexanucleotide (18) repeats were also identified by MISA (Table 4). Among the SSRs, A/T (66.2%) accounted for 99.2% of mononucleotide repeats and AG/CT (8.6%) accounted for 63.4% of dinucleotide repeats, AAC/GTT (2.9%), AAG/CTT (5.7%) and ATC/ATG (3.7%) accounted for 66.8% of trinucleotide repeats (S3 Table). The frequency and distribution of SSRs were dependent on tools, parameters and dataset used[48]. Identification of SSRs provides researchers a very cost-effective way to develop functional markers for various marker-assisted breeding purposes[48]. Primers for these SSRs were also designed using a combination of MISA and Primer3 (S4 Table).

**Table 4:** Statistics of SSRs identified in alfalfa leaf transcriptome.

**Parameter**	**Value**
**SSR identification**	
Total number of sequences examined	192,875
Total size of examined sequences (bp)	165,103,228
Total number of identified SSRs	41,973
Number of SSR containing sequences	33,691 (17.47%)
Number of sequences containing more than one SSR	6,341 (3.29%)
Number of SSRs present in compound formation	2,716 (1.41%)
Frequency of SSRs	One per 3.93 kb
**Distribution to different repeat type classes**	
Mononucleotide	27,991 (66.69%)
Dinucleotide	5,666 (13.50%)
Trinucleotide	7,753 (18.47%)
Tetranucleotide	510 (1.22%)
Pentanucleotide	35 (0.08%)
Hexanucleotide	18 (0.04%)

### Identification of candidate genes related to FD

FD affects the biomass and winter survival rate in alfalfa. We performed gene expression analysis to understand the mechanisms governing fall doramancy. Our results identified 1,841 DE genes were at the May time point (5) between dormant (D) and non-dormant (ND) lines. Likewise, 2,064 genes were different between the genotypes at the September time point (9). A comparison between the two time points in the same cultivars identified 1,780 and 1,652 genes for the D and ND cultivars respectively.

**Fig 8:**
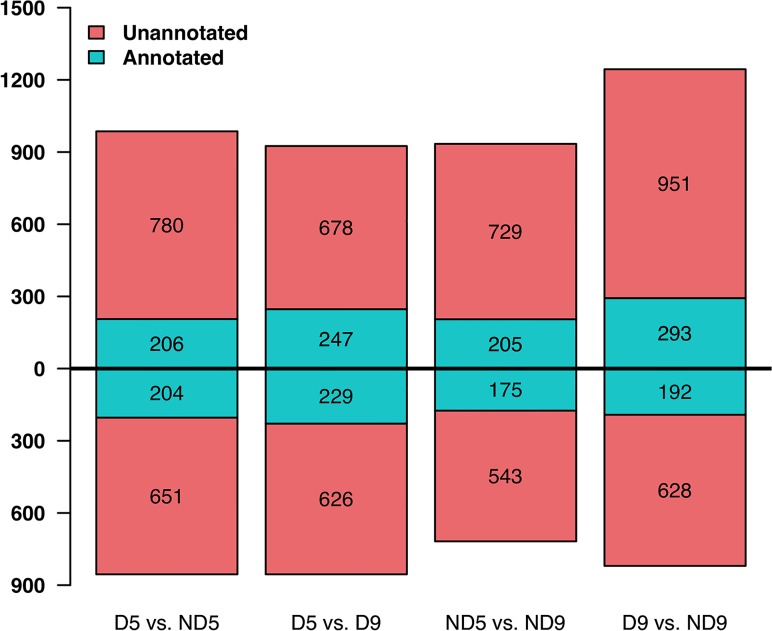
Number of differential expressed (DE) genes identified in our study. DE genes were filtered with at least 2-fold change and adjusted *P* value less than 0.01. Green and red box shows the quantity of annotated and un-annotated DE genes against NCBI NR database (using BLASTX with an E-value threshold of ≤ 1E-05). DE genes above the x-axis were up regulated ones, below were down regulated.

**Fig 9:**
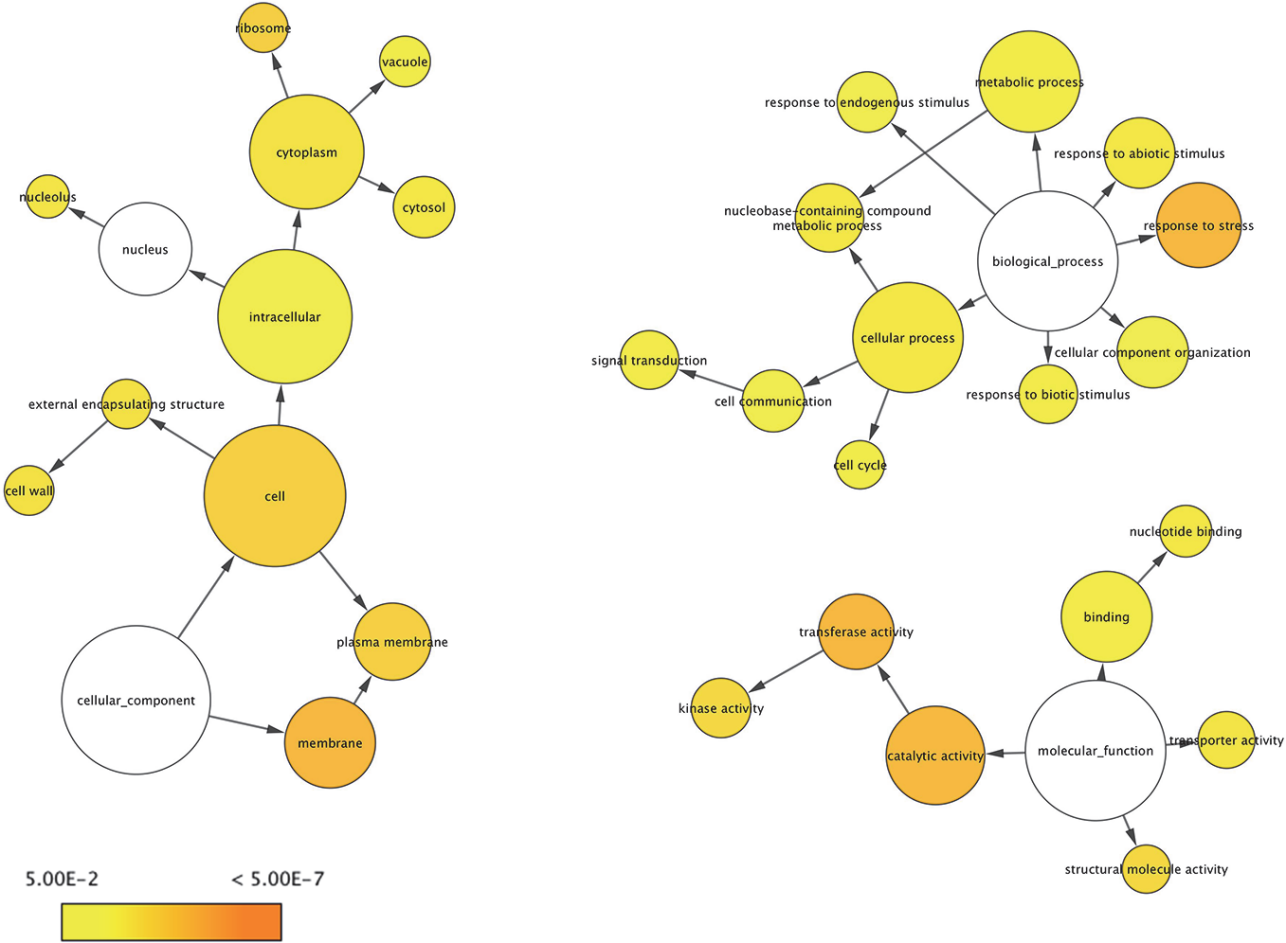
Significantly enriched Gene Ontology categories of DE genes in D5 vs. D9 set using BiNGO. Node size is proportional to the number of transcripts in each category and the significance levels are color coded ranging from 5E-02 to < 5E-07 (white, no significant difference; yellow, *P* = 0.05; orange, *P* < 5E-07).

**Fig 10:**
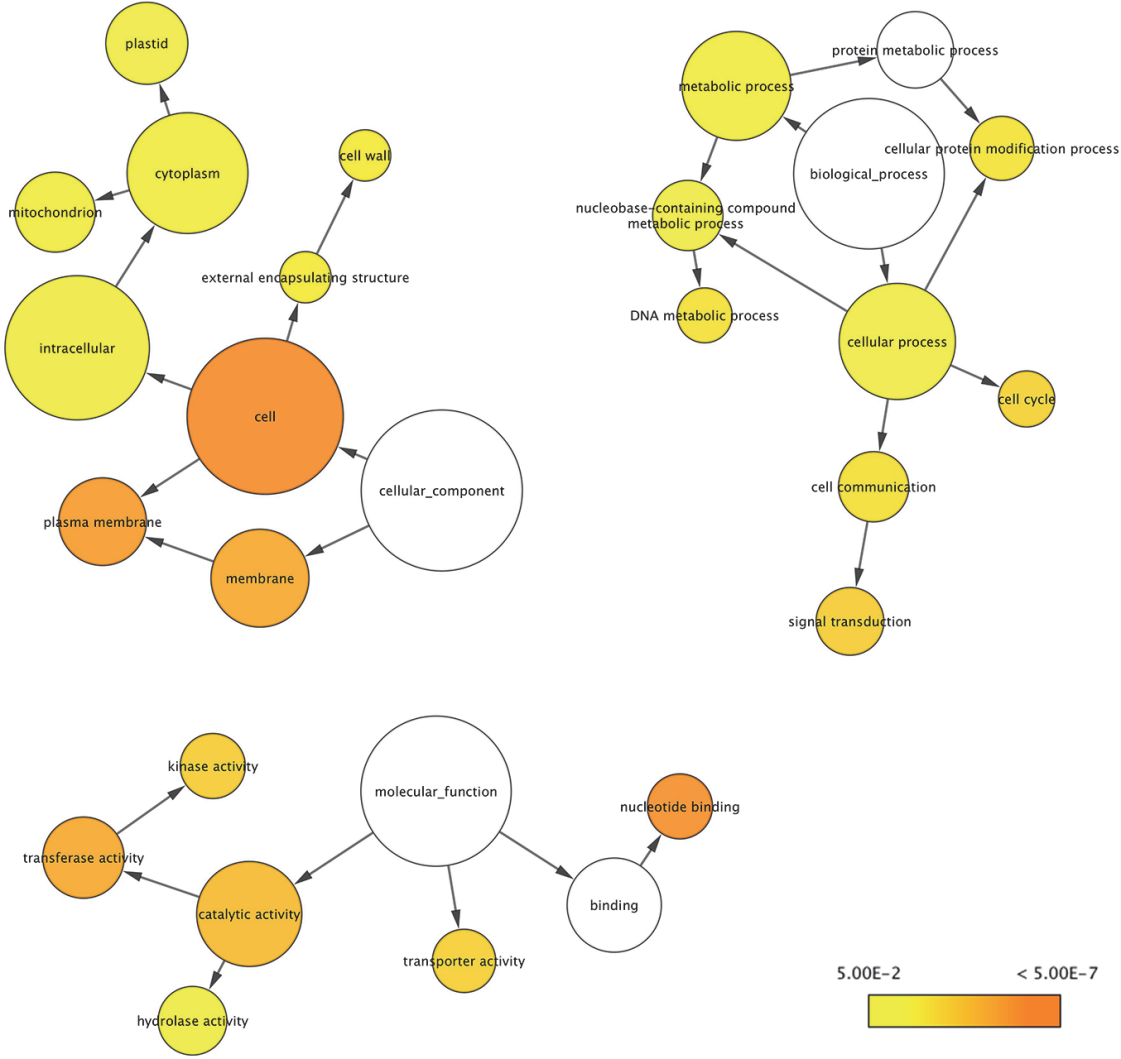
Significantly enriched Gene Ontology categories of DE genes in D9 vs. ND9 set using BiNGO. Node size is proportional to the number of transcripts in each category and the significance levels are color coded ranging from 5E-02 to <5E-07 (white, no significant difference; yellow, *P* = 0.05; orange, *P* < 5E-07).

According to the BLASTX search against the NR database, 410 DE genes in D5 vs. ND5, 485 in D9 vs. ND9, 476 in D5 vs. D9 and 380 in ND5 vs. ND9 had significant hit(s), whereas the others did not get a significant hit. The up- and down-regulated DE genes are also shown in Fig. 8. To find the main functional categories of these DE genes, BiNGO [71] plugin of Cytoscape [72] was used to identify the enriched GO terms. In the D5 vs. ND5 dataset, the catalytic activity, transferase activity and kinase activity in MF category, cell in CC category and response to stress in BP category were the most enriched (S3 Fig.). Structural molecule activity and kinase activity in MF, ribosome, plasma membrane, membrane and cytoplasm in CC were most enriched GO terms in ND5 vs. ND9 set (S4 Fig.). Response to stress in BP, membrane in CC and transferase activity and catalytic activity in MF were the highest enrichment terms in D5 vs. D9 set (Fig. 9). Cell, plasma membrane and membrane in CC, nucleotide binding, transferase activity and catalytic activity in MF and signal transduction in BP were the most enriched GO terms (Fig. 10). Enriched GO terms might provide a clue towards some specific functions operative in alfalfa leaves or other legumes.

Alfalfa standard varieties Maverick (FDC1) and CUF101 (FDC9) were selected as research materials. FD was induced by a combination of shortening day length and falling temperature rather than day length or temperature itself[6,73]. Previous study showed that photoperiod (day length) is a key factor in inducing FD in alfalfa[9]. However, the occurrence of this process is still not clear. Exploring the expression profile of some key genes may be very important for understanding the mechanism of FD in alfalfa. Combined with that fall dormant varieties show FD morphology in late summer or early autumn while non-dormant ones do not show FD morphology[73], two distinct growth seasons (May and September) were chosen. Here, D5 (Maverick in May), ND5 (CUF101 in May) and ND9 (CUF101 in September) are controls in our study while only D9 (Maverick in September) enter FD.

Dormancy in perennial plants has been widely investigated[74]. Leaves, buds, seeds and roots were selected as research objects[18,65,75,76]. Dormancy in plants consists of three types, i.e. paradormancy, endodormancy and ecodormancy[77]. According to [77]andKühn [75], FD in alfalfa is endodormancy. Induction of FD in alfalfa is caused by a combination of shortening day length and falling temperature in late summer or autumn[6]. Previous studies conducted in FD focused on winter hardiness and sugar content in alfalfa roots[14–18]. Horvath [78]showed that perception of day length plays a role in both flowering and endodormancy induction in plants and the genes and processes for perceiving and disseminating day length signals are likely control both the flowering and endodormancy. Both light and temperature influence the timing and impact of circadian clock gene expression[78]. Unfortunately, far less is known about the exact role that the circadian clock plays in endodormancy, but many observations suggest a strong connection[78]. Our previous study about FD focused on phytochromes and hormones[9,79]. With short day length treatment, the biosynthesis of phytochrome B (phyB) and abscisic acid (ABA) was induced[9]. Kühn [75]showed that under short day length treatment, the expression levels of phyA and phyB were very high in endodormancy research in grapevine leaves. Leaves were responsible for perceiving and transmitting of photoperiod (day length) signals[80]. It is possible that the environmental factors that cause endodormancy might act through alterations in the expression of circadian response genes[78], we thought it was a good choice to select leaves of alfalfa as the research objects for FD investigation. We compared four sets of gene expression data, D5 vs. ND5, D5 vs. D9, ND5 vs. ND9 and D9 vs. ND9; each set got more than one thousand DE genes. Due to the lack of rich genomic resources in alfalfa, only a small number of DE genes had been annotated, leaving large amounts of DE genes unknown. Among the four groups in our study, fall dormant type of alfalfa showed FD morphology in September (D9) rather than May (D5), while non-dormant type of alfalfa did not show FD morphology in both May (ND5) and September (ND9). The comparisons of two sets, D5 vs. D9 and D9 vs. ND9 might be more important. Seed and bud dormancy of other plants has been greatly investigated[76, 81–84]. Few studies focused on the molecular level of FD in alfalfa. A previous study in our lab showed that the short day length increases the ABA and phyB concentration and day length is also a key factor for FD in alfalfa[9]. Perry showed that the photoperiod was the control center of dormancy in trees and phytochromes were receptors, which exist in leaves and twigs of many species[84]. Other studies showed that the sugar content was closely associated with winter injury in alfalfa[14–18]. Besides, hormones such as ABA play a key role in regulating response to stress[85], and the expression level has been found to be elevated in some FD alfalfa genotypes or this morphology could be induced by exogenous ABA[86,87].

**Fig 11:**
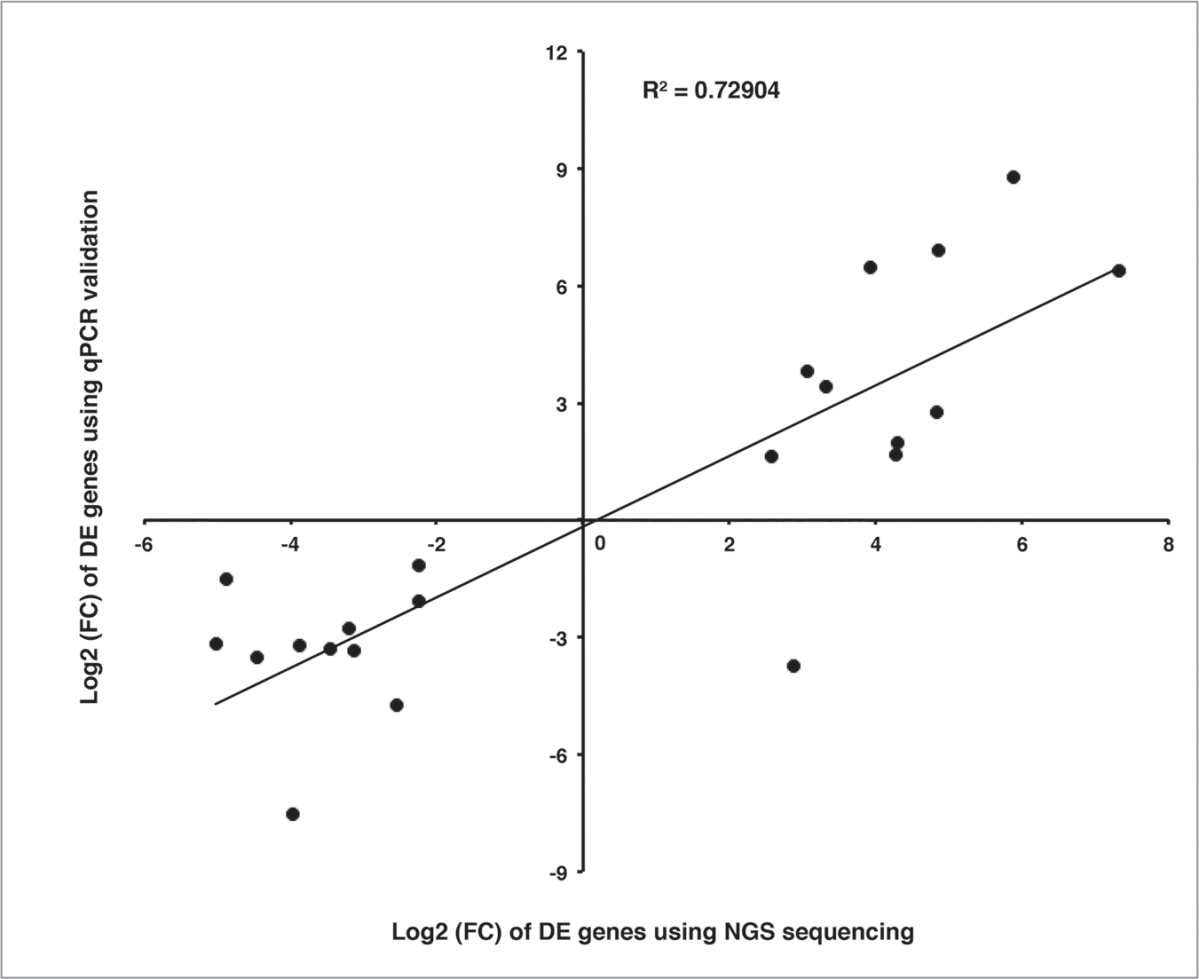
qPCR validation. Differential expressed genes detected through NGS sequencing in our study were validated using qPCR. Twenty-two DE genes were randomly selected for validation. The correlation between NGS sequencing and qPCR was shown.

Although we got more than 1,000 DE genes in each of the four sets, no more than 26.74% (D5 vs. D9, highest across the four sets) of DE genes were annotated against NR database due to the lack of genomic resources in alfalfa. A full list of DE genes in all of the four sets is shown in S5 Table. Previous studies on bud dormancy showed that internal (hormones and sugar) and external signals (light) act together and overlapped signal transduction pathways to regulate the endodormancy, ecodormancy and paradormancy of plants[77]. Hormones like ABA and ethylene have critical roles to induce the senescence of plants[77]. In our D5 vs. D9 set, ethylene-responsive transcription factor RAP2-11 (comp1522760_c0) was up-regulated in D9. Likewise, auxin-induced protein 5NG4 (comp15978_c0) and sugar transporter ERD6-like protein (comp946999_c0) were also up-regulated in D9. TF RAP2-11 belongs to the AP2/EREBP family, which plays important roles such as being key regulators of developmental processes and responding to biotic and environmental stress[88]. Auxin-induced protein 5NG4 was induced by auxin in organs such as roots, hypocotyls and juvenile shoots to form lateral or adventitious roots, and it was down-regulated by auxin; however, the functions in other auxin-mediated processes were not understood[89]. Sugar transporter ERD6-like protein is mainly involved in carbohydrate transportation, monosaccharide transportation, response to ABA and salt stress. In D9, ERD6 was up-regulated might indicate the sugar accumulation in D9 as previously suggested (14–18). Horvath [77]showed that ABA is the primary signal regulating endodormancy and endodormancy is primarily regulated by phytochrome and/or ethylene, sugar could induce the biosynthesis of ABA; the up-regulation of ethylene and sugar would promote the accumulation of ABA thus inhibit the growth in D9 set. Compared to D9, auxin-responsive protein IAA5 (comp33585_c0), auxin-induced protein 5NG4 (comp38464_c0) and auxin response factor (comp705304_c0) were down-regulated in ND9. As we mentioned earlier, D9 showed FD while ND9 did not. Auxin response factors (ARFs) are transcriptional activators and repressors in promoters of early auxin response genes[90]. It is not clear how ARF repressors function in auxin-response gene expression. Tiwari (2003) concluded that these ARF repressors might function on genes that are down-regulated in response to auxin[90]. Auxin-responsive protein IAA5 an Aux/IAA protein was down-regulated in ND9. High levels of auxin increase the degradation of Aux/IAA protein [91] indicating that ND9 had a higher auxin level than D9, which was consistent with the observation that ND9 did not enter FD. Auxin maintains buds dormancy as a result of sufficient auxin provided by apical meristem. Liu (2013) concluded that the auxin function in the ABA-mediated response to seed dormancy/germination inhibition by regulating a seed-specific ABA signaling component [92] in *Arabidopsis*. Auxin is a key factor for elongation during plant stem and root growth. of the stem and root of a plant; however, whether the dormancy in plants is directly regulated by auxins, remains unclear. FD in alfalfa might be induced by shortening day length, which is perceived by the phytochrome or another photoreceptor and these photoreceptors in turn promoted the expression level. At the same time, ethylene and sugar acted to actively promote ABA levels, thus, inhibiting the continuous growth of alfalfa. Dormancy research focused on seeds and buds has been largely investigated, especially in *Arabidopsis*, Poplar and Leafy spurge. However, FD research at the molecular level in alfalfa has not been studied well. We selected leaves as research materials, because FD induction and light signals are perceived and transduced by leaves[79,80,93,94]. Two biological replicates per time point were taken to ensure the transcriptome sequencing and DE gene results. Furthermore, common dormancy-related genes such as *FLOWERING LOCUS T* (FT) and phyA were not found among the DE genes dataset. This could be ascribed to the time points we selected. Perhaps consecutive time points are necessary to fully record the potential genes related to FD in alfalfa. Most DE genes in our study could not be annotated due to the limited availability of alfalfa genomic resources. To validate the gene expression results generated by RNA-seq, twenty-two DE genes across the four sets were randomly selected. Quantitative PCR (qPCR) showed a high correlation (R^2^ = 0.73) of fold change (FC) between NGS and qPCR (Fig. 11).

**Fig 12:**
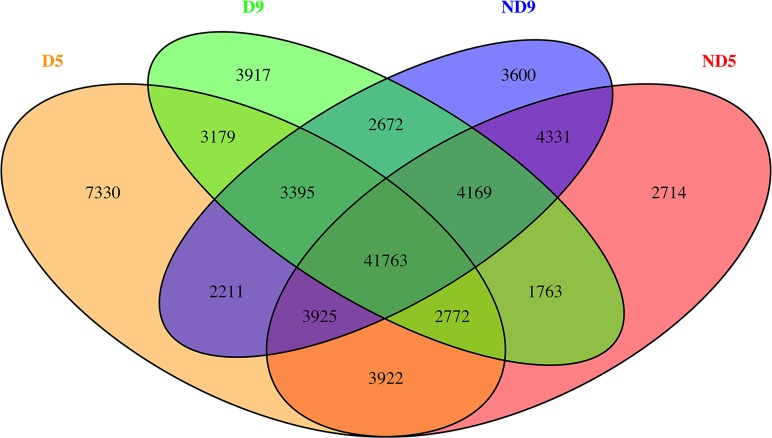
Common and specific genes among or in each of the four conditions in our study. Of the total of 94,700 genes in transcriptome, 41,763 (44.10%) were shared by all of the four sets.D5, ND5, D9 and ND9 for dormant type (Maverick) in May, non-dormant type (CUF101) in May, dormant type in September and non-dormant type in September, respectively.

There were shared and specific genes among or in each of the four conditions in our study. Of the total of 94,700 genes in transcriptome, 41,763 (44.10%) were shared by all of the four sets (Fig. 12). D9 and ND9 specific genes were 3,917 and 3,600, respectively. The main functional categories of D9 and ND9 specific genes were depicted using BiNGO. Response to stress, cell, kinase activity, plasma membrane were the first four enriched GO terms among the D9-specific genes (S5 Fig.). On the other hand, in ND9-specific genes, they were ribosome, structural molecule activity, cytosol and membrane (S6 Fig.). Pathway information was mined using KOBAS 2.0 web service[95]. Enriched pathways are shown in Table 5 and Table 6. According to the KOBAS analysis, Spliceosome, Linoleic acid metabolism, Taurine and hypotaurine metabolism, Phenylpropanoid biosynthesis, Galactose metabolism and Ubiquitin mediated proteolysis were enriched KEGG pathways in D9-specific genes. In ND9-specific genes, Ribosome, ABC transporters and Flavonoid biosynthesis were the enriched pathways. The cut-off *P*-value for KEGG enrichment analysis was 0.05. KOBAS analysis showed enriched pathways of specific genes in D9 and ND9 sets, but the relationship between these pathways and FD is not clear. Further studies and collaborations should be made to uncover these novel, unannotated genes.

**Table 5:** Enriched KEGG pathways in D9-specific genes.

**Term**	***P*-Value**
Spliceosome	5.09E-04
Linoleic acid metabolism	9.94E-03
Taurine and hypotaurine metabolism	0.016
Phenylpropanoid biosynthesis	0.020
Galactose metabolism	0.027
Ubiquitin mediated proteolysis	0.049

**Table 6:** Enriched pathways in ND9-specific genes.

**Term**	***P*-Value**
Ribosome	2.54E-20
ABC transporters	2.21E-03
Flavonoid biosynthesis	8.11E-03

## Conclusions

In conclusion, we sequenced and characterized the leaf transcriptome of alfalfa leaves in two standard varieties, Maverick and CUF 101, at two time points using the Illumina paired-end sequencing technology. Our study generated 192,875 transcripts and characterized some important features of the alfalfa leaf transcriptome in relation to FD including functional annotation, KEGG pathway analysis and identification of TFs. We also identified SSRs, which can be used to develop functional markers. Moreover, we pinpointed some potential DE genes that may relate to FD in alfalfa. Some of the DE genes (annotated and novel) identified in our study exhibited large differences in expression levels. These genes may play important roles in FD in alfalfa. The data generated in this study describes the alfalfa leaf transcriptome in relation to FD, and will provide a great genomic resource for the alfalfa community for FD research. However, further detailed investigations of these DE genes, together with the novel ones, would be very useful in elucidating the mechanism of FD in alfalfa.

## Supporting Information

S1 Fig Isoforms distribution of alfalfa leaf transcriptome. Each alfalfa leaf transcript contains 1 to 16 isoforms. Values above bars indicate the corresponding number of transcripts in each isoform category.
(TIF)

S2 Fig The number of *Arabidopsis*, *Medicago truncatula* and alfalfa transcripts belonging to major TF families. The number of TFs for *Arabidopsis* and *Medicago truncatula* are from Libault et al. (2009).
(TIF)

S3 Fig Significantly enriched Gene Ontology categories of DE genes in D5 vs. ND5 set using BiNGO. Node size is proportional to the number of transcripts in each category, and the significance levels are color coded ranging from 5E-02 to <5E-07 (white, no significant difference; yellow, *P* = 0.05; orange, *P* < 5E-07).
(TIF)

S4 Fig Significantly enriched Gene Ontology categories of DE genes in ND5 vs. ND9 set using BiNGO. Node size is proportional to the number of transcripts in each category, and the significance levels are color coded ranging from 5E-02 to <5E-07 (white, no significant difference; yellow, *P* = 0.05; orange, *P* < 5E-07).
(TIF)

S5 Fig Significantly enriched Gene Ontology categories of D9-specific genes using BiNGO. Node size is proportional to the number of transcripts in each category, and the significance levels are color coded ranging from 5E-02 to < 5E-07 (white, no significant difference; yellow, *P* = 0.05; orange, *P* < 5E-07).
(TIF)

S6 Fig Significantly enriched Gene Ontology categories of ND9-specific genes using BiNGO. Node size is proportional to the number of transcripts in each category, and the significance levels are color coded ranging from 5E-02 to <5E-07 (white, no significant difference; yellow, *P* = 0.05; orange, *P* < 5E-07).
(TIF)

S1 Table Primers used in qPCR validation. (XLSX)

S2 Table Details of KEGG pathways identified using KAAS annotation. (XLSX)

S3 Table Detailed statistics of identified SSRs in alfalfa leaf transcriptome. (XLSX)

S4 Table Primers developed for SSRs in this study using MISA and Primer3. (XLSX)

S5 Table Full list of DE genes in all of the four sets. Unannotated DE genes were left blank in the Protein column.
(XLSX)
