# Supplementary figures and images for: De Novo Characterization of Fall Dormant and Nondormant Alfalfa (Medicago sativa L.) Leaf Transcriptome and Identification of Candidate Genes Related to Fall Dormancy
Source: PLoS One. 2015 Mar 23;10(3):e0122170. doi: 10.1371/journal.pone.0122170 (PMC4370819; doi:10.1371/journal.pone.0122170)

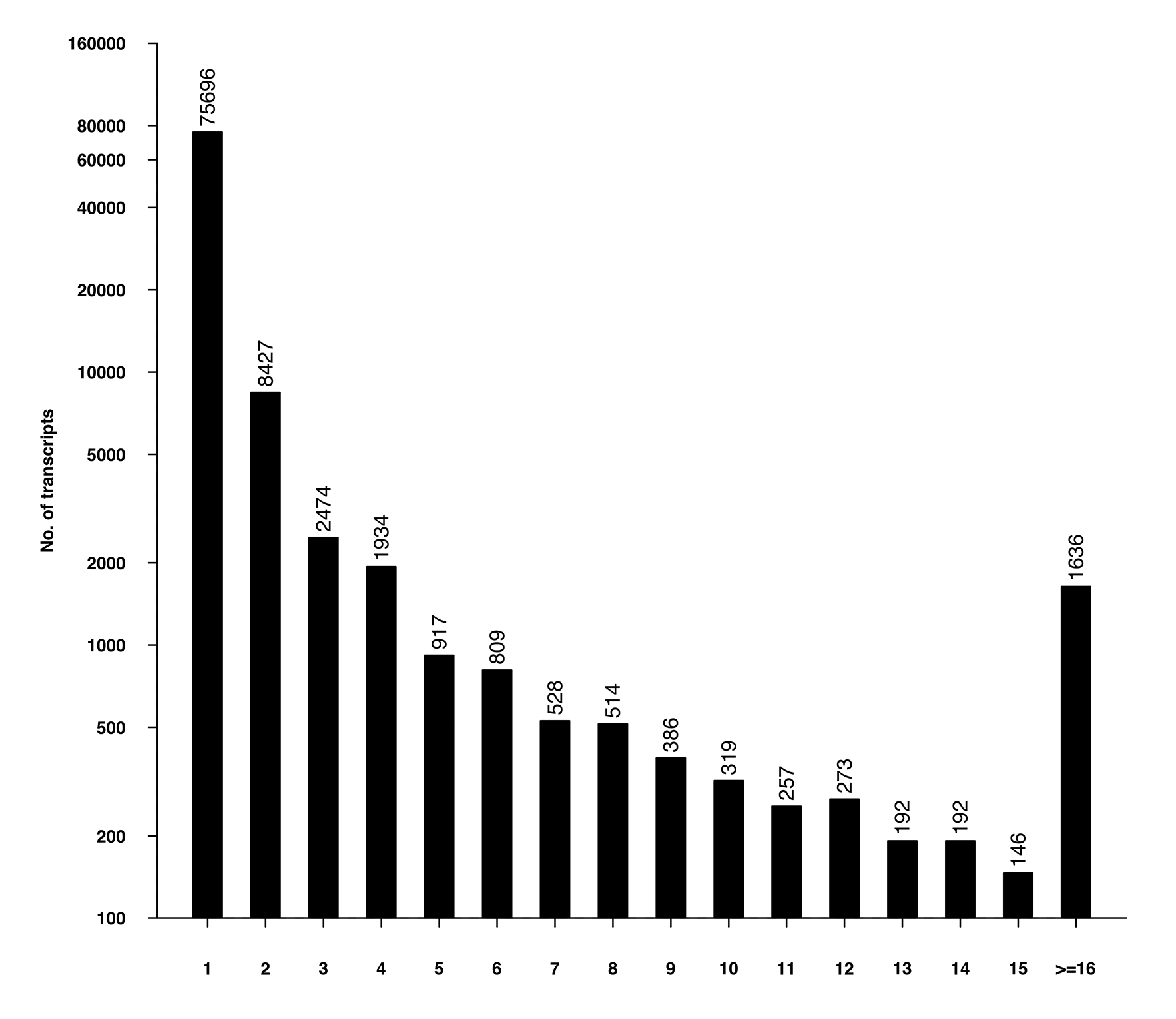

Supplement: S1 Fig — Each alfalfa leaf transcript contains 1 to 16 isoforms. Values above bars indicate the corresponding number of transcripts in each isoform category. (TIF) [file pone.0122170.s001.tif]

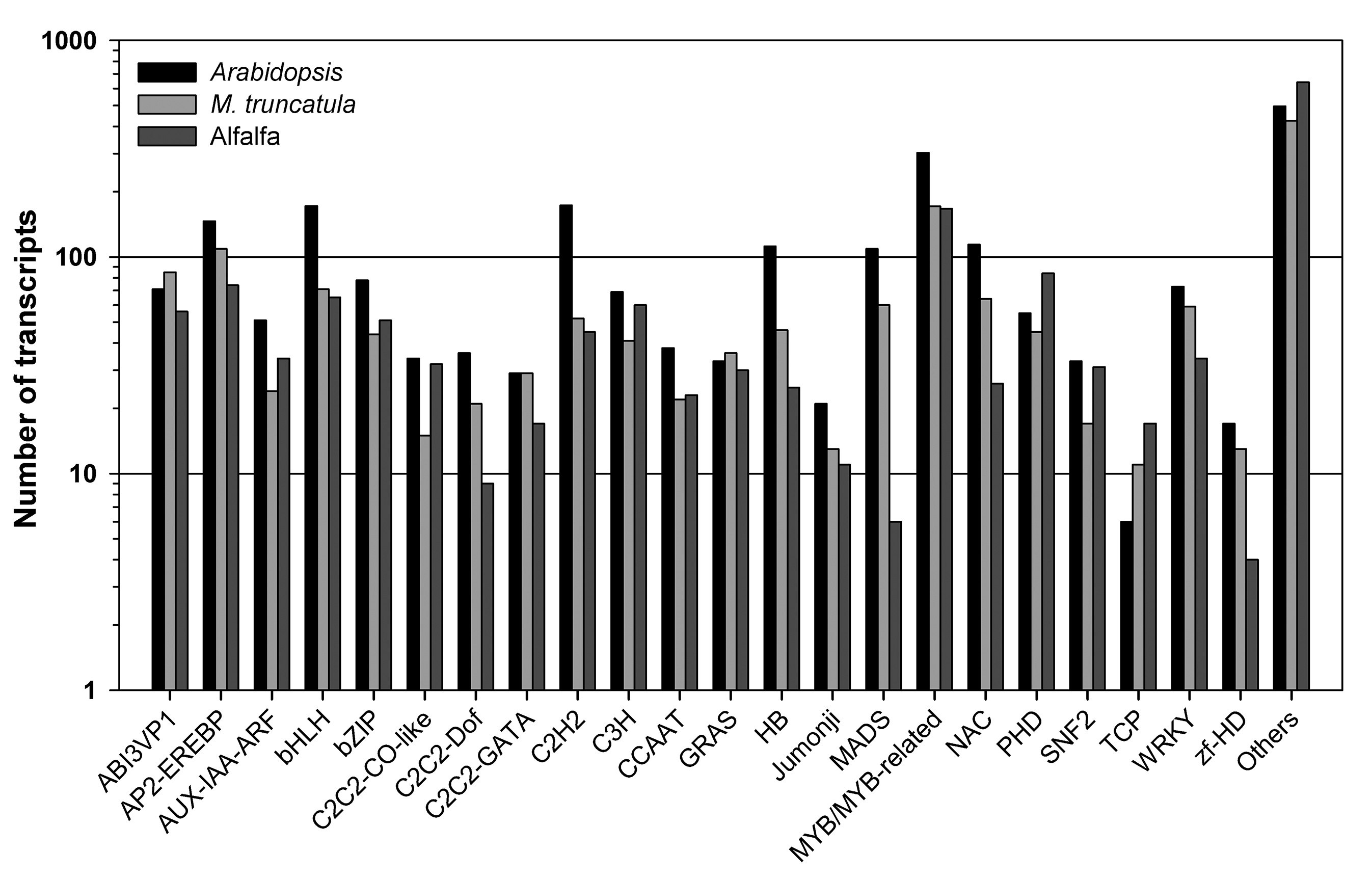

Supplement: S2 Fig — The number of TFs for Arabidopsis and Medicago truncatula are from Libault et al. (2009). (TIF) [file pone.0122170.s002.tif]

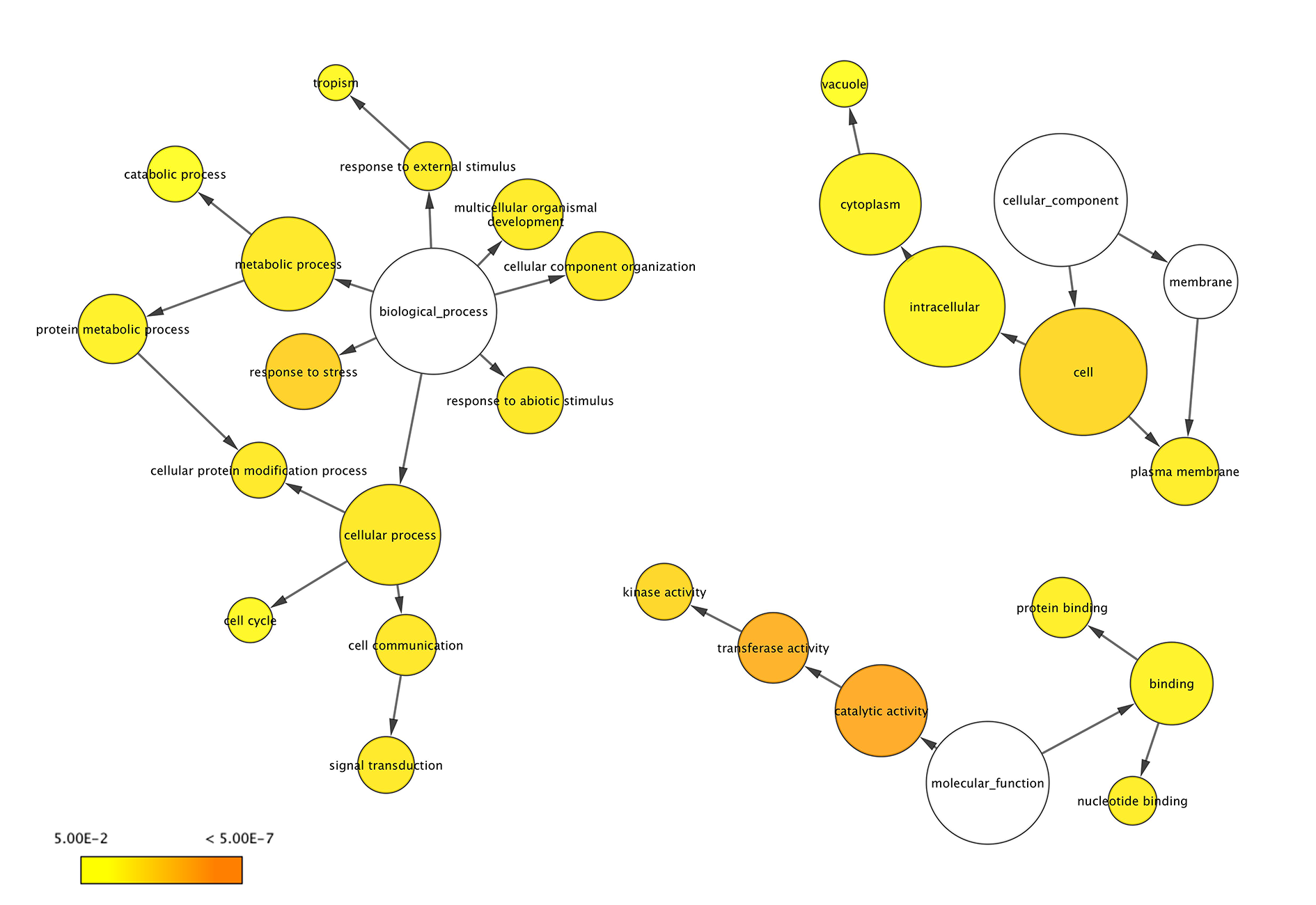

Supplement: S3 Fig — Node size is proportional to the number of transcripts in each category, and the significance levels are color coded ranging from 5E-02 to <5E-07 (white, no significant difference; yellow, P = 0.05; orange, P < 5E-07). (TIF) [file pone.0122170.s003.tif]

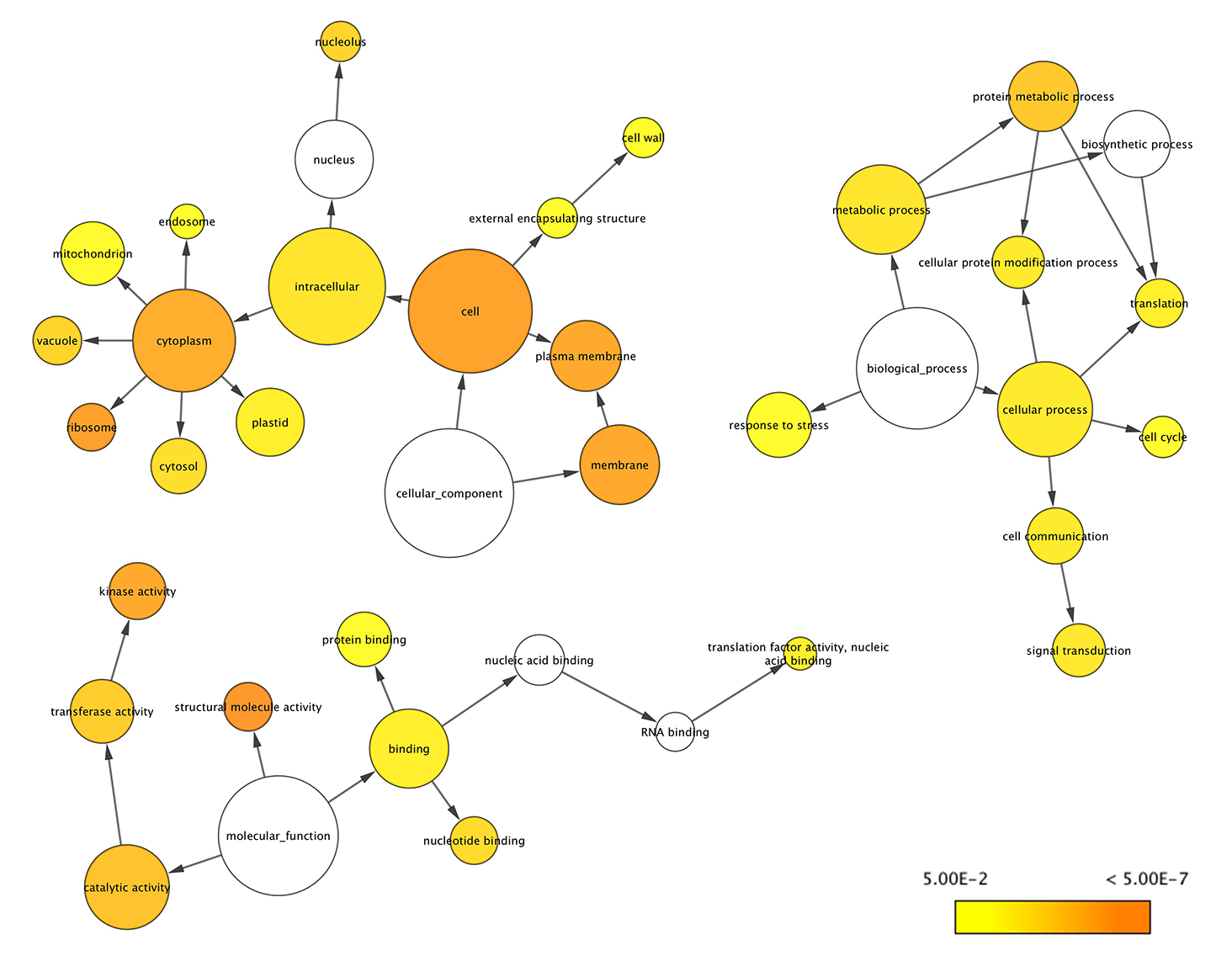

Supplement: S4 Fig — Node size is proportional to the number of transcripts in each category, and the significance levels are color coded ranging from 5E-02 to <5E-07 (white, no significant difference; yellow, P = 0.05; orange, P < 5E-07). (TIF) [file pone.0122170.s004.tif]

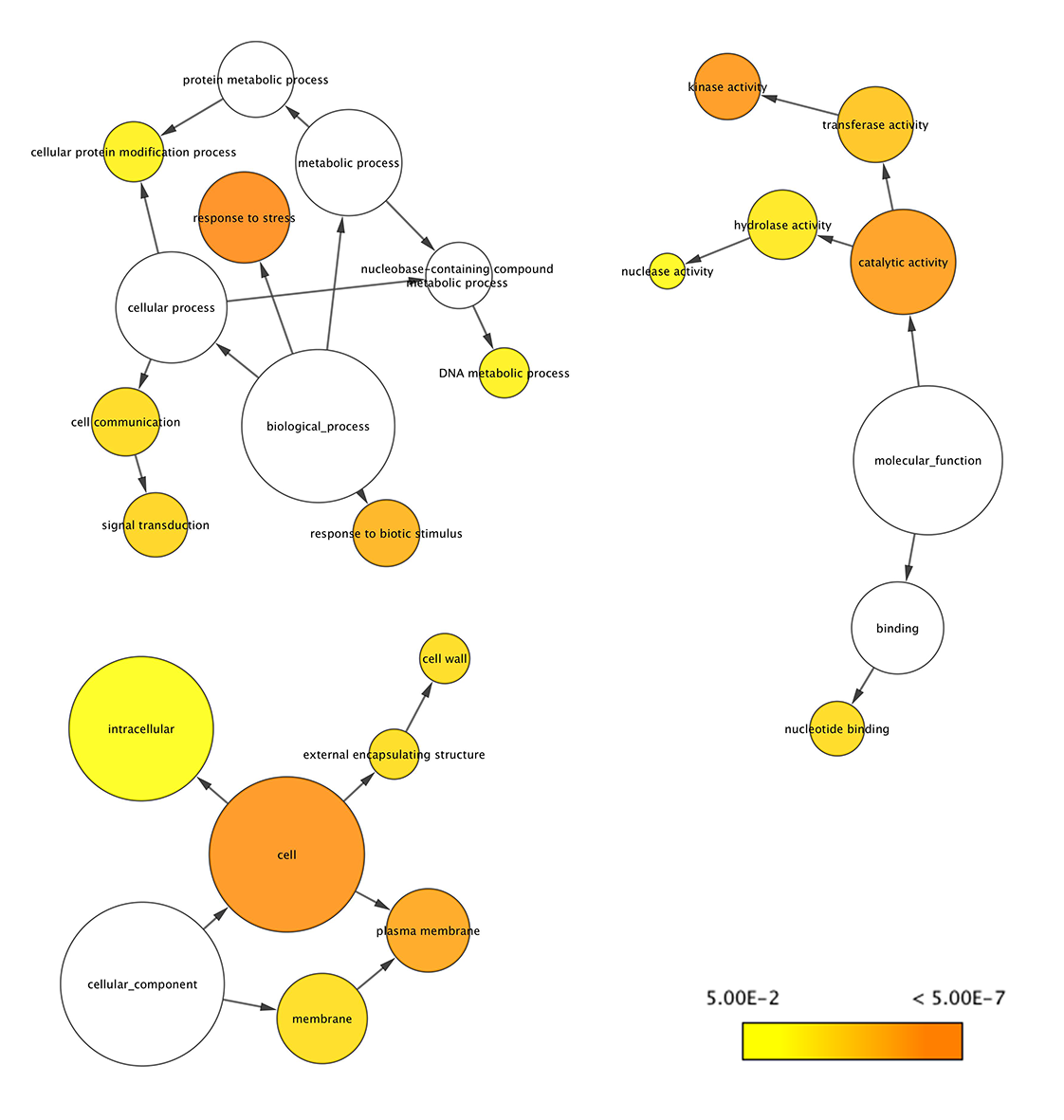

Supplement: S5 Fig — Node size is proportional to the number of transcripts in each category, and the significance levels are color coded ranging from 5E-02 to < 5E-07 (white, no significant difference; yellow, P = 0.05; orange, P < 5E-07). (TIF) [file pone.0122170.s005.tif]

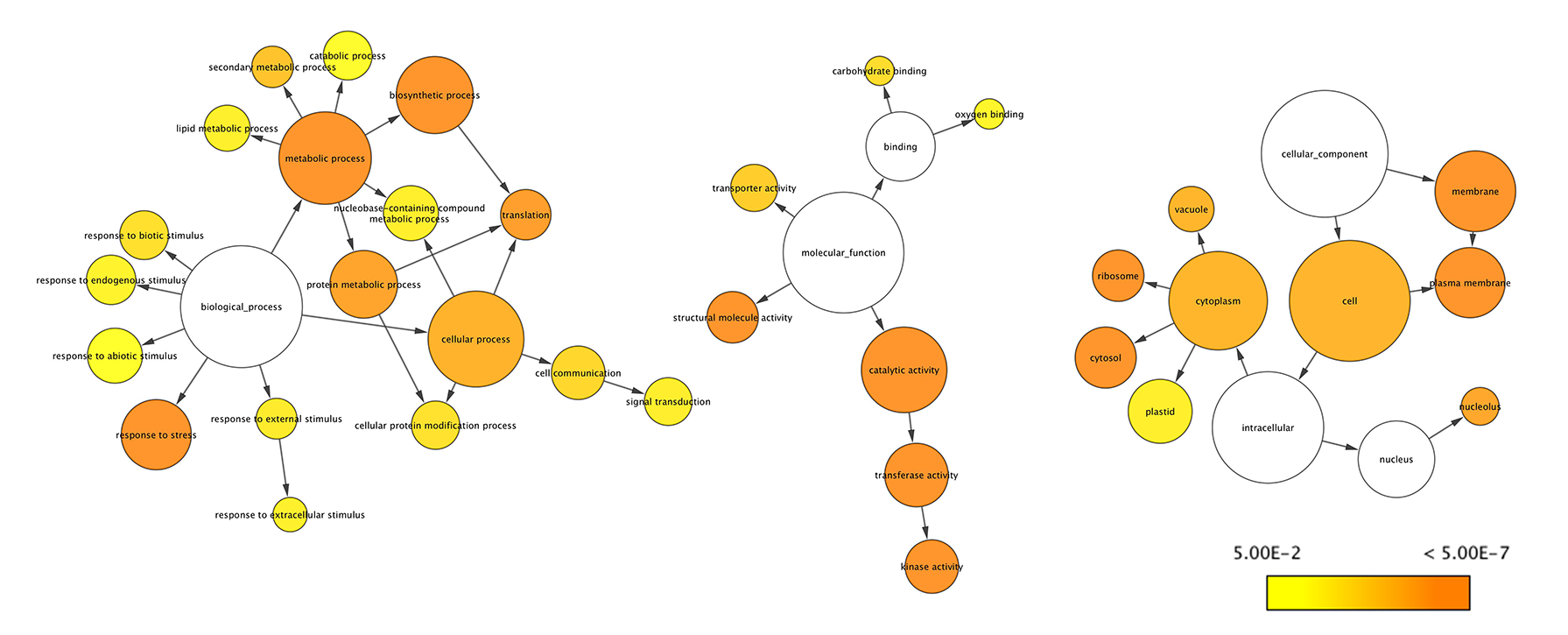

Supplement: S6 Fig — Node size is proportional to the number of transcripts in each category, and the significance levels are color coded ranging from 5E-02 to <5E-07 (white, no significant difference; yellow, P = 0.05; orange, P < 5E-07). (TIF) [file pone.0122170.s006.tif]
